# The association between coding for chronic kidney disease and kidney replacement therapy incidence at CCG-level in England: an ecological study

**DOI:** 10.3399/BJGPO.2024.0171

**Published:** 2025-07-02

**Authors:** Christoph Heinrich Lindemann, James Medcalf, James Hollinshead, Dorothea Nitsch

**Affiliations:** 1 Department II of Internal Medicine and Center for Molecular Medicine Cologne (CMMC), University of Cologne, Faculty of Medicine and University Hospital, Cologne, Germany; 2 UK Renal Registry, UK Kidney Association, Bristol, UK; 3 Department of Cardiovascular Sciences, University of Leicester, Leicester, UK; 4 United Kingdom Department of Health and Social Care, London, UK; 5 Faculty of Epidemiology and Population Health, London School of Hygiene and Tropical Medicine, London, UK

**Keywords:** chronic kidney disease, renal insufficiency, chronic, renal replacement therapy, general practice

## Abstract

**Background:**

With ageing of the population, both prevalence of chronic kidney disease (CKD) and incidence of kidney replacement therapy (KRT) are rising. Existing research suggests that Read-coding for CKD in those affected is associated with better implementation of recommended care and fewer hospitalisations for heart failure.

**Aim:**

To investigate whether coding for CKD is associated with regional KRT incidence in England.

**Design & setting:**

This is an ecological study using the clinical commissioning groups (CCGs) in England as geographical units.

**Method:**

KRT incidence rates were calculated using UK Renal Registry (UKRR) data from January 2019–December 2021. Data on the percentage of uncoded CKD patients (PUCP), who had laboratory evidence of CKD but lacked a diagnostic code, were obtained from the CVDPREVENT Audit, a national audit that extracts routinely held GP data. Data on confounders and acute kidney injury (AKI) mortality as a marker for population frailty were obtained from CVDPREVENT and the UKRR, respectively. Poisson models assessed the association between PUCP and KRT incidence.

**Results:**

After adjusting, the PUCP was non-linearly associated with KRT incidence, with the CCGs in the lowest PUCP quintile having a lower KRT incidence than the others. There was evidence that this association was more pronounced in CCGs with high AKI mortality compared with CCGs with low AKI mortality.

**Conclusion:**

At the geographical level in England, the data suggest that the prevalence of not having formally diagnosed CKD is non-linearly associated with a higher KRT incidence rate, especially in areas with a high AKI mortality.

## How this fits in

Owing to the long period without symptoms, chronic kidney disease (CKD) is often recognised (too) late and not treated adequately. It has been shown that adding a Read code for CKD in the GP setting when there was laboratory evidence of CKD was associated with a higher quality of care for these patients and led, for example, to fewer cardiovascular diseases. This study shows that adding a Read code may also be associated with lower kidney replacement therapy (KRT) incidence at a geographical level in England. These associations could be used to project future regional KRT needs.

## Introduction

Chronic kidney disease (CKD) is characterised by a progressive loss of kidney function. Patients with CKD are at a much higher risk of developing other life-threatening diseases owing to the crucial role of kidneys in maintaining bodily homeostasis.^1,2^ In the UK, the estimated prevalence of CKD stages 3–5 in the adult population is around 5%–7%.^3,4^ Notably, CKD is associated with costs of 1.45 billion pounds or 1.3% of all NHS expenditure in the UK.^5^

Early diagnosis of CKD, and subsequent holistic treatment, can slow down the loss of kidney function and prevent complications. In the UK, there are pay-for-performance targets in primary care for appropriately diagnosing and managing chronic diseases, including hypertension, diabetes, and CKD stages 3–5.^6^ Despite this, up to 30% of patients with laboratory evidence of CKD were not appropriately Read-coded in primary care, with large differences between individual practices.^3,7–9^

Adding a Read code for CKD to those with biochemical evidence of CKD stages 3–5 is a marker of GP awareness, and is positively associated with better cardiovascular risk management in those with CKD, as well as better coding of hypertension and diabetes and negatively associated with deprivation.^7,8,10–12^ At the population level, the percentage of patients with laboratory-based CKD who have been appropriately coded in primary care can be considered a quality marker for the care of patients with CKD. In prospective analyses, patients with a CKD stage 3–5 Read code had fewer cardiovascular and heart failure hospitalisations, AKI episodes, and lower mortality than those uncoded with the same level of kidney function.^9,13^

A small subset of people with CKD will progress to kidney failure (KF), at which point a kidney replacement therapy (KRT) in the form of dialysis or transplantation is required. In England, there are large regional differences in the crude KRT incidence rates, ranging from 85 to 214/1 000 000 in different areas of England. Even after standardisation by age and sex, there was still a large variability in the rates.^14^ Data are conflicting on whether CKD prevalence is associated at country-level with KRT incidence.^15^ This mismatch of regional CKD prevalence with regional KRT incidence may be explained by the start of KRT not always being planned; sometimes patients start dialysis after developing severe acute kidney injury (AKI). AKI is more common in people with CKD, and especially in those who are not coded for CKD.^3^ AKI is also associated with a higher risk of multimorbidity, death, or serious diseases, especially in more severe stages.^16^ It has been shown that frail people are more likely to die from an AKI episode.^17^ Against this background, AKI mortality at the population level can be seen as a marker for the underlying population frailty.

Notably, the COVID-19 pandemic had a massive impact on the KRT incidence, with a decrease of 8.6% from 2019 to 2020 in the UK after a slight upward trend beforehand.^18^ Despite COVID-19 being associated with a high risk of developing AKI, which can lead to a significant decrease in kidney function, the recent drop in KRT incidence can potentially be explained by older frail patients dying during the pandemic. As a result, trends in KRT incidence become unpredictable owing to the lack of sufficient longitudinal data following the COVID-19 pandemic.

Owing to the structural, financial, and personnel efforts regarding KRT, the planning process requires estimates of the future development of KRT incidence. Before the COVID-19 pandemic, these figures could be extrapolated from previous years.^19^ However, this is no longer possible owing to the massive impact of the pandemic on the CKD population.^18^

We carried out a cross-sectional ecological study to investigate the association between the percentage of uncoded CKD patients (PUCP), readily available from an ongoing primary care audit, and KRT incidence (2019–2021) at the regional level of clinical commissioning groups (CCGs) in England. If there is an association at the regional level, it may be possible to project future regional KRT incidences using longitudinal data on these metrics. Because the data for KRT incidence include the pandemic period, it is further hypothesised that the association between PUCP and KRT incidence at CCG level in England varies depending on the frailty of the population within a given CCG, using AKI mortality at CCG level as a proxy for frailty.

## Method

### Study design

This ecological cross-sectional study focuses on the 106 CCGs in England, which serve as the geographical units. CCGs were NHS bodies that organised health care in the respective regions during the period in which the data for this study was collected and the data were analysed in line with how they were generated in clinical care at that time. As part of the restructuring in 2022, the CCG geographical areas are now referred to as Sub-ICB (integrated care boards) Locations.

The study included only adult patients aged ≥18 years. Only pre-existing and aggregated data at CCG level were used.

### Data sources

Population denominator data to calculate prevalences or incidences were obtained from the Cardiovascular Disease Prevention (CVDPREVENT) Audit.

The outcome was the incidence of KRT in the CCGs, defined as initiating dialysis or kidney transplantation as the first treatment without prior dialysis. The data were provided by the UK Renal Registry (UKRR). Patients who received dialysis for fewer than 90 days were omitted. The coverage was 100% across all CCGs. Owing to the small number of events in each CCG, the aggregated numbers for the 3 years from January 2019–December 2021 were used to calculate the average yearly incidence.

The exposure was the percentage of patients in the CCGs who met the laboratory criterion for CKD stage 3 or higher but had no GP-recorded CKD code. The CVDPREVENT Audit provided the data up to March 2021.

Covariates were included to assess potential confounding or mediation of the association between exposure and outcome. All covariate data were used at CCG level. An overview and more information on all data used and their sources can be found in Supplemental Table 1 and the Supplemental Methods.

There were no missing data.

### Conceptual framework

**Figure 1.**
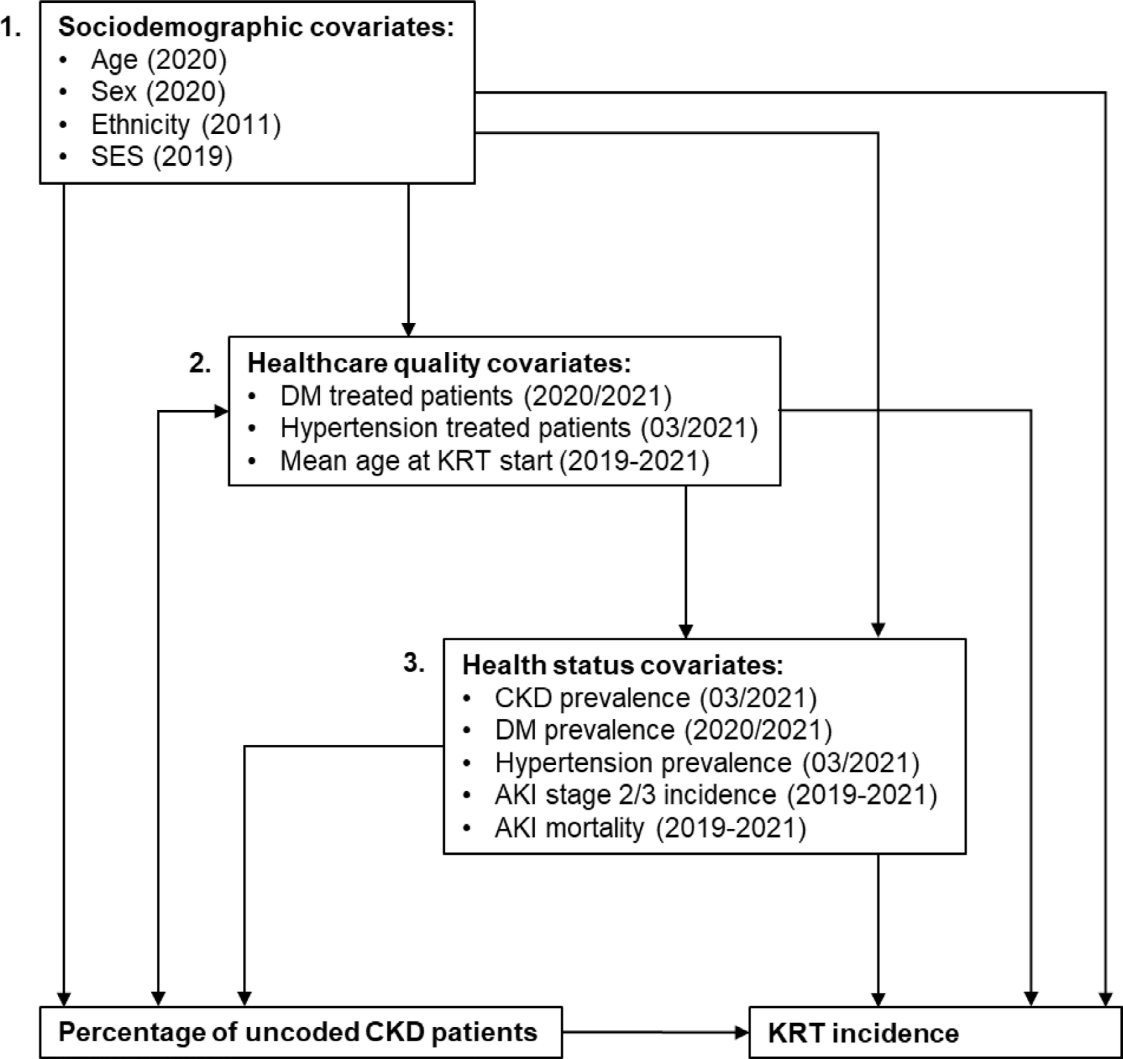
Hierarchical conceptual framework with three hierarchies of covariates influencing the association between PUCP and KRT incidence. In parentheses is the time up to which or period when the data were collected. AKI = acute kidney injury. CKD = chronic kidney disease. DM = diabetes mellitus. KRT = kidney replacement therapy. SES = socioeconomic status

A hierarchical conceptual framework was developed to assess the association between exposure and outcome (Figure 1). At CCG level, covariates could be grouped into sociodemographic, healthcare quality**,** and health status covariates and accordingly, three hierarchies were considered for multivariable modelling.

### Analysis strategy

KRT incidence was categorised into quintiles with care taken to ensure that CCGs were evenly distributed across the quintiles.

Correlation matrices were drawn for the exposure and each covariate group of the conceptual framework to assess associations between the exposure and the covariates. If both variables were normally distributed, Pearson’s r was calculated; otherwise, Spearman’s ρ.

Associations were assessed using Poisson regression owing to the number of people starting KRT being a count variable. Since the outcome variable was KRT incidence (a rate), an offset term was included to allow for the differing population sizes of each CCG (denominator data). It was assumed that the CCGs were independent and could be given equal weight. Non-linearity between exposures and covariates and KRT incidence was assessed using scatter plots and quadratic terms. Only CKD prevalence showed a non-linear relationship with KRT incidence. Therefore, CKD prevalence and the Index of Multiple Deprivation (IMD) score (relative score) were transformed into categorical variables in quintiles. Similarly, a categorical variable was defined for the exposure in addition to the continuous to investigate potential non-linear associations.

Following the hierarchical conceptual framework, a sequence of three Poisson models was created. Some variables were strongly correlated, which could have led to multicollinearity after including them in the models. That was assessed by comparing the crude and the fitted models’ root-mean-square errors (RMSE).

It was tested whether the association between PUCP and KRT incidence was modified by the a priori-defined effect modifier AKI mortality, using a simplified full model for which AKI mortality was recoded to a binary variable (low or high fraction) based on an equally large population in each fraction. Evidence of effect modification was assessed using likelihood ratio tests (LRTs).

For all Poisson models, LRTs were conducted to assess the evidence for a difference in rates.

Owing to the slight change in the boundaries between three CCG pairs between 2019 and 2021, these pairs were merged for sensitivity analysis. A second sensitivity analysis was done using the Office for National Statistics (ONS) population denominator data.

Statistical analysis was performed in R Studio (version 2023.06.0).^20^ Maps were created using QGIS (version 3.22).^21^

## Results

### Description of KRT incidence and PUCP

**Figure 2.**
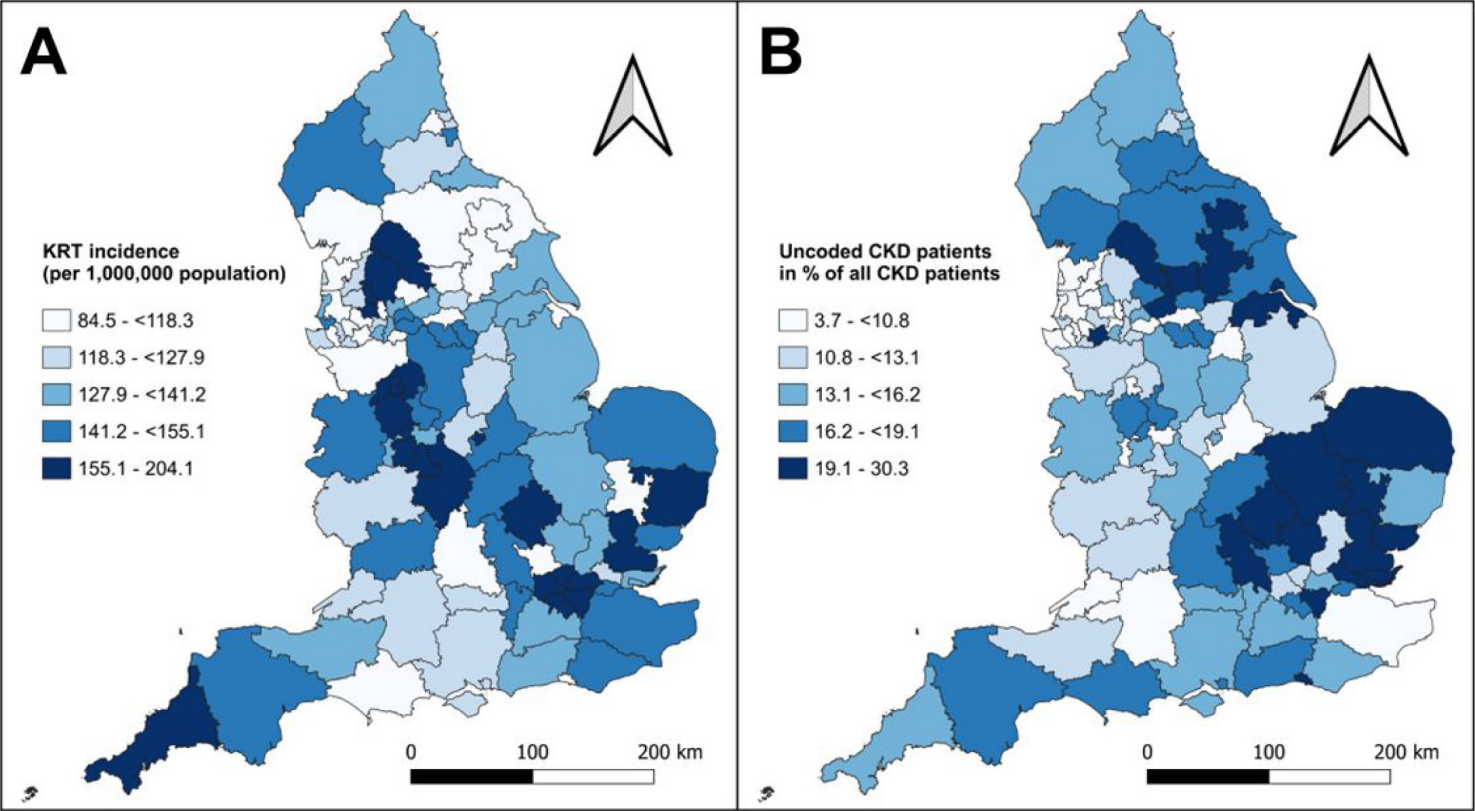
Spatial distribution of (A) KRT incidence rates and (B) PUCP at CCG level in England. Boundary data: Office for National Statistics^34^ *n* = 106. CCG = clinical commissioning group. CKD = chronic kidney disease. KRT = kidney replacement therapy. PUCP = percentage of uncoded CKD patients.

From January 2019–December 2021, 20 409 adults in England started KRT and the crude overall incidence rate was 141.30/1 000 000, ranging from 84.54/1 000 000 to 204.1/1 000 000 in the different CCGs (Figure 2A). The mean PUCP across all CCGs was 15.47% (standard deviation [SD] 5.55), ranging from 3.7%–30.3% (Figure 2B).

### CCG characteristics

Across KRT quintiles, the median mean population age of all CCGs was 49.53 years (interquartile range [IQR] 47.37–50.79) and lowest in the highest quintile at 47.34 (IQR 45.33–50.25) (Table 1). The median male-population percentage of all CCGs was 49.55 (IQR 49.11–50.03). There was a trend present for the median percentage of non-White population: in the lowest KRT incidence quintile, the median percentage was only 4.05% (IQR 2.73–10.23) and increased steadily to 21.70% (IQR 5.60–31.60) in the fifth quintile. A similar trend was observable for diabetes prevalence with a higher prevalence in CCGs with a higher KRT incidence. These trends could also be observed at the level of the individual CCGs (Supplemental Figure 1).

**Table 1.** CCG characteristics overall and by KRT incidence quintiles

CCG KRT incidence quintile		Overall	Quintile 1	Quintile 2	Quintile 3	Quintile 4	Quintile 5
Number of CCGs		106	22	21	21	21	21
CCG KRT incidence per 1 000 000 population		84.5 to 204.1	84.5 to <118.3	118.3 to <127.9	127.9 to <141.2	141.2 to <155.1	155.1 to 204.1
**Outcome**							
	KRT incidence per 1 000 000 population, mean (SD)	135.39 (22.35)	106.15 (9.18)	123.38 (2.51)	133.92 (4.01)	146.75 (4.09)	168.12 (11.85)
**Exposure**							
	Uncoded CKD patients in % of all CKD patients, mean (SD)	15.47 (5.55)	15.87 (5.59)	11.40 (4.59)	17.42 (6.63)	15.53 (3.80)	15.57 (5.35)
**Covariates**							
**Sociodemographic covariates**							
	Mean population age in years, median (IQR)	49.53 (47.37–50.79)	49.55 (48.67–50.52)	49.20 (47.33–50.35)	49.53 (48.57–51.51)	49.92 (48.78–51.31)	47.34 (45.33–50.25)
	Male population in %, median (IQR)	49.55 (49.11–50.03)	49.57 (49.16–49.86)	49.62 (49.20–50.20)	49.29 (48.88–49.97)	49.31 (48.77–49.73)	50.03 (49.53–50.48)
	IMD score, median (IQR)	21.35 (17.12–29.48)	20.80 (16.40–27.08)	23.60 (18.30–29.60)	19.40 (15.50–25.20)	20.40 (18.80–27.10)	23.00 (18.20–30.90)
	Non-White population in %, median (IQR)	6.35 (3.30–12.12)	4.05 (2.73–10.23)	4.60 (3.00–9.80)	5.90 (3.10–9.70)	6.90 (4.00–9.80)	21.70 (5.60–31.60)
**Healthcare quality covariates**							
	Hypertension treatment goal achieved in %, mean (SD)	60.12 (3.63)	60.65 (3.52)	60.68 (3.25)	59.70 (4.27)	60.05 (4.21)	59.49 (2.87)
	Diabetes treatment goal achieved in %, mean (SD)	61.52 (2.32)	61.92 (2.15)	60.92 (2.49)	62.11 (2.80)	61.04 (1.97)	61.59 (2.09)
	Mean KRT starting age in years, mean (SD)	61.76 (2.50)	61.67 (2.29)	61.32 (2.71)	61.90 (2.42)	62.66 (2.04)	61.24 (2.92)
**Health status covariates**							
	CKD prevalence in %, mean (SD)	4.77 (1.21)	4.69 (0.98)	4.99 (1.08)	4.60 (1.10)	5.09 (1.28)	4.10 (1.14)
	Hypertension prevalence in %, mean (SD)	16.76 (2.32)	16.52 (2.54)	16.74 (2.26)	16.55 (2.23)	17.15 (1.66)	15.70 (2.81)
	Diabetes prevalence in %, mean (SD)	7.46 (0.90)	6.99 (0.94)	7.43 (0.80)	7.52 (0.86)	7.55 (0.55)	7.80 (1.12)
	AKI stage 2–3 incidence per 1000 population, median (IQR)	3.51 (3.05–3.88)	3.43 (2.94–3.89)	3.58 (3.35–4.00)	3.71 (3.06–3.81)	3.42 (3.16–3.68)	3.51 (2.65–4.08)
	AKI mortality in % of AKI patients, median (IQR)	24.53 (23.15–25.53)	24.17 (22.23–-25.34)	24.42 (23.56–25.14)	25.04 (24.11–25.67)	24.51 (23.09–25.81)	24.98 (23.68–25.55)

AKI = acute kidney injury. CCG = clinical commissioning group. CKD = chronic kidney disease. IMD = Index of Multiple Deprivation. IQR = interquartile range. KRT = kidney replacement therapy. SD = standard deviation

### Correlations of the PUCP and covariates

**Figure 3.**
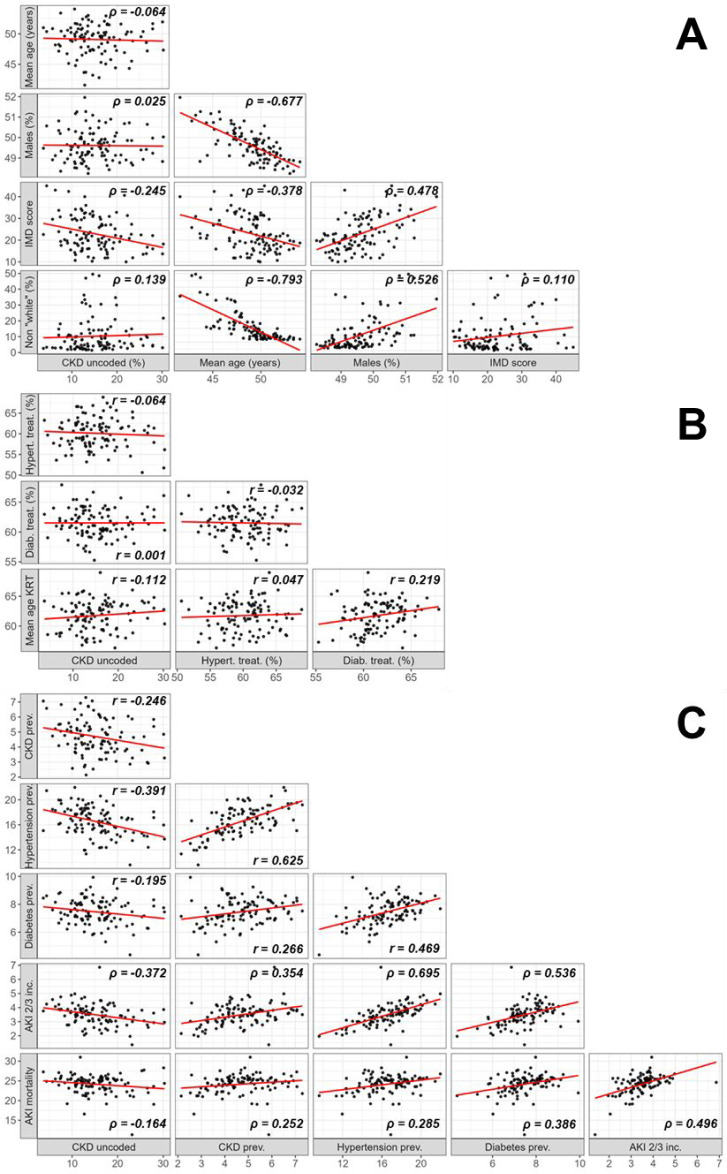
Cross-correlation scatter plots of the (A) PUCP and sociodemographic covariates, (B) the PUCP and healthcare quality covariates, and (C) the PUCP and health status covariates. If both variables were normally distributed, the correlation coefficient Pearson’s r and otherwise Spearman’s ρ are presented for each correlation. *n* = 106. AKI = acute kidney injury. CKD = chronic kidney disease. Diab. treat = diabetes treatment goal achieved. Hypert. treat = hypertension treatment goal achieved. IMD = Index of Multiple Deprivation. inc = incidence. KRT = kidney replacement therapy*.* pop = Population. prev = prevalence

A crude negative correlation was observed between the PUCP and IMD (*ρ* = −0.245) (Figure 3A). Between the sociodemographic covariates, strong negative correlations were found with the mean population age: a higher mean population age was associated with a lower male population (*ρ* = −0.677), a lower IMD score (*ρ* = −0.378), and a lower non-White population (*ρ* = −0.793). Conversely, a higher percentage of male population was positively correlated with a higher IMD score (*ρ* = 0.478) and a higher non-White population (*ρ* = 0.526).

There was no evidence of any crude correlations between the PUCP and one of the healthcare quality covariates (Figure 3B). The healthcare quality covariates showed a positive correlation between the percentage of patients with diabetes who had achieved their treatment goal and the mean KRT starting age (*r* = 0.219).

A higher PUCP was crudely correlated with lower CKD prevalence (*r* = −0.246), hypertension prevalence (*r* = −0.391), and AKI stage 2–3 incidence (*ρ* = −0.372) (Figure 3C). Between the health status covariates, correlations were positive: a higher CKD prevalence was strongly correlated with a higher hypertension prevalence (*r* = 0.625) and a higher AKI stage 2–3 incidence (*ρ* = 0.354). A higher AKI stage 2–3 incidence was also strongly correlated with a higher hypertension prevalence (*ρ* = 0.695), a higher diabetes prevalence (*ρ* = 0.536), and a higher AKI mortality (*ρ* = 0.496).

The correlation coefficients and corresponding *P* values for the correlations of all variables, including KRT incidence, can be found in Supplemental Table 2.

### Univariable and multivariable analysis

Univariable Poisson regression, showed no evidence of an association between the crude PUCP (as a continuous variable) and KRT incidence (rate ration [RR] 1.000, 95% confidence interval [CI] = 0.997 to 1.003) (Table 2). However, when included as a categorical variable, there was very strong evidence (*P*<0.001) for a difference in the crude KRT incidence rates, indicating a non-linear association, with the highest rate in the second quintile, 22% higher than in the first.

**Table 2.** Univariable (crude) and multivariable Poisson regression models of the association between the PUCP and the KRT incidence

		Rate ratio (95% CI)	*P* value
**Crude**			
	Uncoded CKD patients in % (continuous)	1.000 (0.997 to 1.003)	0.886
	Uncoded CKD patients in % (quintiles)		
	Lowest	1	<0.001
	2	1.221 (1.167 to 1.277)	
	3	1.123 (1.074 to 1.174)	
	4	1.075 (1.029 to 1.123)	
	Highest	1.099 (1.051 to 1.149)	
**Model 1** ^a^			
	Uncoded CKD patients in % (continuous)	1.002 (0.999 to 1.005)	0.149
	Uncoded CKD patients in % (quintiles)		
	Lowest	1	0.062
	2	1.033 (0.982 to 1.087)	
	3	1.051 (1.003 to 1.102)	
	4	1.000 (0.954 to 1.048)	
	Highest	1.044 (0.996 to 1.093)	
**Model 2** ^b^			
	Uncoded CKD patients in % (continuous)	1.003 (1.000 to 1.006)	0.093
	Uncoded CKD patients in % (quintiles)		
	Lowest	1	0.360
	2	1.020 (0.969 to 1.075)	
	3	1.030 (0.982 to 1.082)	
	4	1.000 (0.953 to 1.050)	
	Highest	1.037 (0.990 to 1.087)	
**Model 3** ^c^			
	Uncoded CKD patients in % (continuous)	1.004 (1.000 to 1.008)	0.029
	Uncoded CKD patients in % (quintiles)		
	Lowest	1	0.030
	2	1.080 (1.021 to 1.143)	
	3	1.061 (1.009 to 1.116)	
	4	1.035 (0.982 to 1.090)	
	Highest	1.077 (1.020 to 1.138)	

^a^Adjusted for sociodemographic covariates. ^b^Adjusted for sociodemographic and healthcare quality covariates. ^c^Adjusted for sociodemographic, healthcare quality and health status covariates*. P* values are calculated using likelihood-ratio tests. *n* = 106. CI = confidence interval. CKD = chronic kidney disease

After adjusting for all covariates (Model 3), an association between the PUCP as a continuous variable and KRT incidence was found (RR 1.004, 95% CI = 1.000 to 1.008, *P* = 0.029). An association was also present when using the PUCP as a categorical variable (*P* = 0.030). The difference in rates between the first and the other quintiles ranged from a 3.5% higher rate in the fourth and an 8% higher rate in the second quintile. The RMSE consistently decreased across all models, indicating that the models benefitted from including the additional covariates.

### Effect modification

**Figure 4.**
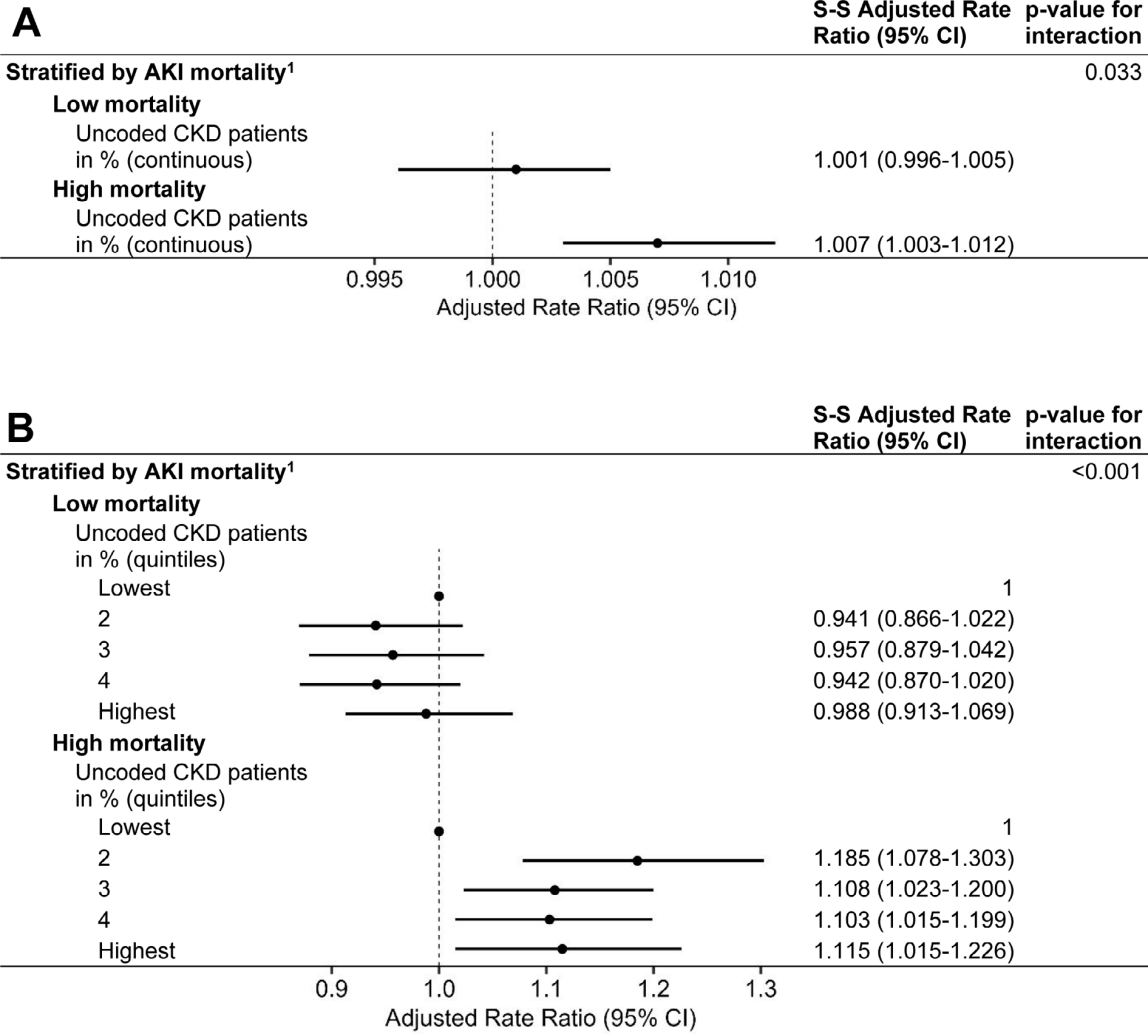
Stratum-specific adjusted rate ratios for the effect of AKI mortality (low or high) on the association between PUCP and KRT incidence.^1^Adjusted for sociodemographic, healthcare quality, and health status covariates. The effect of AKI mortality is assessed using PUCP as a (A) continuous and (B) categorical variable. *P* values are calculated using likelihood-ratio tests. *n* = 106. AKI = acute kidney injury. CI = confidence interval. CKD = chronic kidney disease. S-S = stratum specific

Considering the PUCP as a continuous variable, there was some evidence (*P* = 0.033) for effect modification: in CCGs with low AKI mortality, there was no evidence of an association with KRT incidence (RR 1.001, 95% CI = 0.996 to 1.005), whereas in CCGs with high AKI mortality, a strong association was found (RR 1.007, 95% CI = 1.003 to 1.012) (Figure 4).

Using the PUCP as a categorical variable, there was very strong evidence (*P*<0.001) for effect modification by AKI mortality; in CCGs with low AKI mortality, there was no evidence of an association with KRT incidence. However, in CCGs with high AKI mortality, KRT incidence rates from the second up to the fifth quintile were higher than in the first quintile, with the highest rate in the second quintile, 18% higher than in the first one.

Similar results of the effect of AKI mortality on the association between PUCP and KRT incidence were found when using the merged CCG data or population or denominator data from the ONS (Supplemental Table 3 and 4).

## Discussion

### Summary

In this study PUCP was non-linearly associated with the KRT incidence at CCG level in England, with very strong evidence for an effect modification of this association by AKI mortality in that CCGs with higher AKI mortality were observed to have an association of PUCP with KRT, while no such association was observed for CCGs with low AKI mortality.

### Comparison with existing literature

The PUCP at CCG level was used in this study as a marker for the care quality for patients with CKD. The positive association between successful CKD-specific treatments and a slowed progression of CKD has already been described.^22^ A lower KRT incidence rate might also be driven by more older patients and patients with multimorbidity consciously deciding against starting KRT. Given that such a decision is usually made with the treating doctor, it can be considered part of good care.

The large regional differences in KRT incidences and the PUCP have already been described in previous work, where other risk factors, such as ethnicity and diabetes prevalence, were identified.^23,24^ Differences were further aggravated during the COVID-19 pandemic.^18,25,26^

The effect modification of the association between the PUCP and KRT incidence by AKI mortality showed that care for patients with CKD might be critical in frail populations. It has been shown that more than 70% of patients reaching KF are frail.^27^ Furthermore, higher frailty incidence rates have been observed in patients with a faster decline in kidney function, which might have been additionally accelerated by COVID-19 infections.^28^ Finally, it has been shown that frailty is an independent risk factor for KF and the need for KRT.^29^ This might explain why better care in this population is particularly associated with a lower KRT incidence.

An explanation of the non-linear associations found might be explained by wide range of percentages the first quintile covered. Therefore, the lower KRT incidence in this quintile could be driven by CCGs with a very low PUCP and presumed excellent care. However, using quintiles is a rough method to assess for more flexible non-linear associations higher than quadratic terms.

### Strengths and limitations

An ecological study was chosen because the PUCP is a structural factor, representing practices or areas, and its association with KRT incidence in the population was of interest.^30^ Using data from only one country reduces the problem of systematic differences, which can be observed when using data from different countries. Routinely collected, aggregated data with broad coverage ensures that the entire population is included, reducing selection bias. However, 5% of GP practices did not participate in the CVDPREVENT Audit, which might have led to measurement error of the PUCP.^31^ However, inferences of causality should not be concluded from ecological studies. They can only create hypotheses that should be tested using individual-level data. This study compared CCGs in England but not at different time points. It was not possible to calculate incidences for the individual years owing to the sparseness of the data. Thus, it was not possible to depict the development of KRT incidence over time, nor to show any variability over this period. However, the COVID-19 pandemic had a massive impact on health care and KRT incidence, and trends established using data pre- and post-COVID-19 would not have been meaningful.^25,26,32^

Although the final model adjusted for various covariates, unmeasured confounding owing to covariates not included cannot be ruled out. Ideally, frailty should not be defined using proxies but the validated Fried frailty criterion;^33^ however, these data are not available at population level.

At the time of the study, data on ethnicity was only available from the 2011 Census. It is therefore possible that these data do not entirely represent ethnicity in the period 2019–2021.

CCGs, on the basis of which the analysis was carried out, have been replaced by ICBs and therefore no longer exist. However, for the analysis it was important to use the groupings corresponding to the data generation process to determine the impact of variation in care at the primary care level on RRT rates. Furthermore, we wanted to preserve the granularity that is crucial for signal detection in ecological studies such as ours, which would have been lost if we had lumped the date further into larger ICBs. Finally, CCGs continue to exist in the form of ICBs, so they can still be seen as an organisational structure, albeit in a different role to the CCGs.

### Implications for research and practice

This study shows at an ecological level that there is indirect evidence that conscious awareness of CKD at GP level, in the form of appropriate coding, may be associated with lower KRT incidence rates, particularly in areas with a high proportion of frail people. This can have a significant impact on the ICB budget, which has to cover RRT provision. Especially in the wake of the COVID-19 pandemic, which has disrupted health care and affected KRT incidence. PUCP follow-up data may be the first way to estimate future KRT needs, as extrapolating incidences using previous years is no longer possible after the pandemic.

However, a single ecological study’s results do not justify changes in the current policy. Further research, including individual-level studies, is needed to strengthen the evidence found and guide policymakers on which aspects of care quality are crucial for avoiding KRT.

## Data

All data can be shared on reasonable request to the corresponding author.
